# Palliative care nursing and moral coherence: Early responses to MAiD as hastened death

**DOI:** 10.1177/09697330261465860

**Published:** 2026-07-30

**Authors:** Marianne Sofronas, Dimitri Létourneau, Lisa Chan, Mary Ellen Macdonald, David Kenneth Wright

**Affiliations:** 1 School of Nursing, Faculty of Health Sciences, 6363 University of Ottawa, Ottawa, ON, Canada; 2 Palliative Care and Nursing Ethics Hub, Centre for Research on Health and Nursing, 6363 University of Ottawa, Ottawa, ON, Canada; 3 Faculty of Nursing, 5622 Université de Montréal, Montreal, QC, Canada; 4 Department of Medicine, 3688 Dalhousie University, Halifax, NS, Canada

**Keywords:** qualitative research, palliative care, end of life issues, professional ethics

## Abstract

**Background:**

Since its inception over half a century ago, a defining attribute of palliative care philosophy has been an insistence that this approach intends to neither hasten death nor prolong life. A commitment to facilitate a ‘natural’ end-of-life trajectory is ubiquitous in palliative care discourse. However, as an increasing number of jurisdictions around the world legislate to allow assisted death, palliative care providers are having to re-evaluate the relevance of this commitment for the relational ethics of their practice.

**Research Aims and Participants:**

In this paper, we explore how a sample of Canadian specialist palliative care nurses reflected on this longstanding disciplinary ethic of never intending to hasten death amidst evolving professional and ethical responsibilities to support people who explore and choose medical assistance in dying (MAiD).

**Research Design:**

A qualitative analysis of semi-structured interviews with Canadian speciality palliative care nurses, conducted shortly after Canada legalized MAiD. We present an interpretive description through a framework of moral reflection; moral reflection consists of moral intuition, relational ethics, and moral coherence.

**Ethical Considerations:**

Approval was granted from the Research Ethics Board of the University of Ottawa. Informed consent was obtained in writing and confirmed verbally at the time of each interview. We removed identifiers such as participant roles and geographic locations from interview quotes to protect confidentiality.

**Findings:**

Across the different components of moral reflection, palliative care nurses negotiated their ethical positions amidst changes to end-of-life care.

**Conclusions:**

Moral reflection and striving for moral coherence are ongoing processes; the goal is an engagement with moral plurality. As new jurisdictions consider and legalize MAiD, revisiting the early moral reflections of palliative care nurses can help nurses currently wrestling with similar ethical questions.

## Introduction

Since its inception, palliative care as a philosophy and clinical speciality has been grounded in core principles, including a commitment to relieving physical, psychosocial and spiritual suffering; affirming life and allowing patients to live fully until death; recognizing death as a normal process; and neither intending to hasten death nor prolong life.^1^ Over the past few decades, MAiD has been legalized under certain conditions in Australia, Austria, Belgium, Canada, Colombia, Ecuador, Germany, Italy, Luxembourg, the Netherlands, New Zealand, Portugal, Spain, Switzerland, Uruguay, and some jurisdictions in the United States. The passing of MAiD legislation and the accompanying societal debates have disrupted the stability of palliative care principles, causing palliative care clinicians to re-examine their relevance and practice implications. Prior to MAiD legalization, requests for hastened death were generally interpreted as signals of intolerable suffering requiring intensified palliative care.^2^

Early literature from jurisdictions with established assisted dying legislation such as Belgium and Oregon suggests that palliative care nurses navigate complex, nuanced ethical tensions, weighing the limits of symptom management against honouring and upholding patient autonomy.^3,4^ While experiencing and reflecting on personal discomfort, a large proportion of hospice providers prioritized therapeutic alliance and non-abandonment when patients pursued assisted dying.^5^ Across jurisdictions, health professionals situated themselves across a spectrum of ethical positions.^6^

In Canada, the legal landscape for assisted death has seen many developments over the past decade. Legislation for MAiD was first implemented in Quebec in 2015, and in the rest of the country in 2016, offering access to medically assisted death for adults who were at the end of life. In 2021, legislation expanded to include a two-track system extending eligibility for people whose death was not reasonably foreseeable and introducing a waiver of final consent for those who risked losing capacity. In 2025, the Quebec government integrated advanced requests for MAiD into provincial law. The Canadian legislation extended eligibility for MAiD to people whose sole underlying medical condition is a mental illness, with patients eligible as of March 2027.^7–9^

The relationship between the palliative care principle of never intending to hasten death nor prolong life and MAiD presents tensions for clinicians, professions, and institutions. Competing master narratives –dominant professional storylines that shape group identity– exist concerning the compatibility of the two.^10^ One narrative positions MAiD as antithetical to the palliative care ethos, calling for a strict boundary to protect palliative care’s commitment to a natural death. A competing narrative situates MAiD within palliative care’s goals of alleviating suffering and supporting patient autonomy. In Canada following legalization, this tension explains why institutions providing palliative care carefully considered whether and how to participate in assisted death. Palliative care nurses contended with uncertainty over how they should respond when patients inquire about MAiD.^10–17^ These studies reveal how nurses navigated role ambiguity, moral distress, and the emotional and professional weight of proximity to assisted death. While some nurses turned to conscientious objection to protect their moral integrity, others had ethical justifications for integrating MAiD into their care practices. From within a rich landscape of perspectives, palliative care nurses have expressed their commitment to ensuring that all patients, including those who are eligible for MAiD, receive high-quality end-of-life care. This includes support in setting and achieving goals of care, expert symptom relief, promotion of psychosocial and existential wellbeing, attention to grief, and the creation of meaningful experiences that reflect peoples’ hopes, preferences, and values.^18^

Palliative care organizations have also taken positions in these debates. The International Association of Hospice and Palliative Care highlighted their opposition to MAiD unless universal access to palliative care was ensured, and argued that palliative care units should not oversee and administer MAiD.^19^ This statement was endorsed by over 190 organizations worldwide, including the Canadian Hospice and Palliative Care Association, which reaffirmed its position in 2023, arguing that hospice palliative care and MAiD are distinct practices.^20^

Nurses assume a relational and moral responsibility to orchestrate a good death; this takes on new dimensions in the context of MAiD, as the person being cared for often remains cognitively and biologically intact until the moment of death.^21^ Existing literature has examined this relationship between palliative care nursing philosophy and MAiD as a negotiation of palliative care nurses’ moral and professional identity and moral agency,^22,23^ a process of inhabiting an in-between space of uncertainty, reflection, and sensemaking,^15^ and as shaped by relational and sociopolitical influences involving clinicians and family members.^24^ This body of work highlights the ethical complexity of MAiD in palliative care, while leaving unresolved how the disciplinary commitment to never intending to hasten death is negotiated in clinical practice.

While the legislative landscape in Canada has expanded since MAiD was introduced in 2016, the initial moral reflections of palliative care nurses during those early years remain highly relevant. Establishing a historical baseline provides context to understand trajectories of moral reflection when disciplinary philosophies intersect with legislative change. It can also support nurses in other jurisdictions who are encountering new MAiD legislation.

## Aim

In this paper, we explore, through a framework of moral reflection,^11^ how Canadian specialist palliative care nurses reflected on this ethic of never intending to hasten death nor prolong life, amidst what was, at the time, a transformation of professional and ethical responsibilities to support and care for people who explore and choose MAiD. This inquiry is part of a larger qualitative study examining how participants navigated their professional and moral identities following MAiD legislation.^22^ Our purpose in revisiting these interviews with this focus, is to help inform the moral reflections of nurses in other jurisdictions where debates are ongoing and/or where legislative change toward legalization is new or imminent.

## Framework

Pesut, Thorne and Greig^11^ conceptualized nurses’ decision-making regarding participation in MAiD as a process of moral reflection, rather than simply a matter of adherence to professional codes of ethics or deference to individual moral values. For these authors, moral reflection comprises moral intuition, relational ethics, and moral coherence.

Moral intuition refers to a sense of moral rightness shaped over time through emotional, cognitive, and sociocultural processes, grounded in how individuals live in relation to others within a community.^11^ Moral intuition is more likely to change when there is significant social interaction around a phenomenon, ensuring personal and community coherence.^25,26^ This reinforces the collective nature of intuition and the complementarity of moral intuition and moral reasoning.

Relational ethics considers how taking one moral position over another impacts nurses’ relationships with patients, families, and the broader community. Pesut and colleagues^11^ emphasized the centrality of nurses’ relationships with patients based on genuine caring, mutual respect, and accompaniment. They leaned on Wurzbach^27^ who saw the nursing role in assisted death as an ethic based on ‘staying with’ patients, involving presence, active engagement, non-judgement, and non-abandonment.

Moral coherence involves integrating new moral challenges in ways that remain consistent with one’s broader moral commitments, often through contemplation, comparison with similar cases, and ethical reasoning.^11,25,28^

While this framework was initially developed in a discussion paper examining conscientious objection, its utility is not limited to exploring oppositional stances. Rather, it captures ethical complexity and supports moral reflection and imagination as nurses make sense of their decision to participate in MAiD – or not.^11^ The different components of moral reflection allowed us to hold multiple and sometimes conflicting considerations, professional obligations and relationships, as nurses navigated moral agency, relational practice, and honouring the needs and wishes of patients and families.

## Methods

Through the framework of moral reflection,^11^ we explored the impact on palliative care nurses of having one’s moral intuition challenged, how they considered the impact of MAiD on their practice and their relationships, and how, irrespective of specific viewpoint, they moved towards moral coherence.

We analyzed data from interviews with Canadian specialist palliative care nurses about their ethical understandings of MAiD, conducted in the recent aftermath of MAiD’s legalization as an end-of-life care option. Participants were recruited with announcements circulated through the Canadian Palliative Care Nursing Association and the Canadian Nurses Association. Between 2018 and 2019, twenty-two nurses from across Canada participated in individual interviews lasting 46–122 min (average 83 min). Table 1 summarizes participant demographics.

**Table 1.** Participant demographics (*n* = 22).

Average age in years	52 (range 30–69)
Gender	21 women; 1 man
Regions represented	Western Canada (9)
	Eastern Canada (8)
	Atlantic Canada (5)
	**
	Urban/suburban (10)
	Rural (3)
	Both urban and rural (9)
Average years worked as a nurse	27 (range 8–47)
Average years worked in palliative care	14 (range 3–33)
Highest education	Diploma or Associate degree in Nursing (7)
	Bachelors in Nursing (9)
	Master’s or other postgraduate degree (6)
National certification in hospice palliative care nursing	Yes (16)
	No (6)
Setting of palliative care practice	Hospice or palliative care unit (8)
	Acute care hospital (2)
	Community (7)
	Works across settings (5)
Role in palliative care	Direct care nurse in community or hospital (5)
	Clinical nurse specialist (2)
	Nursing administrator (6)
	Other (e.g. palliative care consultant, resource nurse, coordinator, educator) (9)
Employment status	Full time (17)
	Part time (5)

The interviews (conducted by the third author and transcribed verbatim) began by asking participants what being a palliative care nurse means to them. They described their values as palliative care nurses, and the implications of these values for their feelings about the legalization of MAiD in Canada. Participants were asked how MAiD affected their practice context (their workplace and the larger field of palliative care nursing), and about specific experiences caring for patients requesting and/or receiving MAiD. Participants were also asked how they integrated assisted death within the ethos of palliative care, specifically regarding the disciplinary ethic of neither hastening death nor prolonging life.

Some participants identified themselves as conscientious objectors to MAiD, with a variety of viewpoints. Others supported MAiD as a practice but did not feel it aligned with the philosophy of palliative care. Others still felt it did not belong in clinical care at all. Several nurses were supportive of the practice and offered reflections on debates in their workplace and speciality.

An analysis of how these participants negotiated their moral identity, at the metaphorical fault lines of palliative care and MAiD, is published elsewhere.^22^ Guided by Interpretive Description,^29^ the current analysis considers more closely the palliative care principle of neither hastening death nor prolonging life. A methodology designed for applied health disciplines to generate insights from subjective experiences, Interpretive Description allowed us to move beyond recording participant viewpoints and understand how meaning was constructed as nurses navigated the introduction of MAiD to their practice. Interpretive Description aligned nicely with the moral reflection framework; moral reflection provided a structure to organize the ethical dimensions of nurses’ experiences, while Interpretive Description enabled a deeper understanding of how these moral reflections were mapped onto the realities of palliative care nursing practice. First, syntheses were prepared for each interview by the first author, outlining moral reflections and ethical reasoning articulated by participants. Guided by an inductive approach, transcripts were reviewed for pertinent themes by the first two authors, ensuring these themes emerged directly from the data. Once these themes were identified, the moral reflection framework was introduced as an interpretive lens. Relevant passages were categorized by theme, then transposed onto the framework to deepen our understanding of how nurses navigated ethical tensions. Iterative team discussions allowed us to interrogate the data, refine interpretations, and resolve ambiguities. 

## Ethical Considerations

Ethics approval was granted from the Research Ethics Board of the University of Ottawa (#H02-17-17). Nurses who were interested in participating in the study contacted the research team for information. Informed consent was obtained in writing and confirmed verbally at the time of the interview. Participants were free to decline answering any questions and could withdraw at any time. Given the sensitivity of the topic, we balanced fidelity to participant voices with a commitment to confidentiality by not associating individual quotes with nurses' roles or geographic locations. 

## Findings

### A) Intuitional Responses

For some participants, the philosophy of neither hastening death nor prolonging life aligned with their personal values and beliefs. These included beliefs about the sanctity of life, as well as what constitutes a good life and a good death.

### Religious Beliefs Regarding Death and Dying

Religious beliefs about the sacredness of human life underpinned some participants’ stance that MAiD was incompatible with palliative care. They identified the principle of not hastening death as the bedrock for their practice, and articulated that participating in MAiD inflicted spiritual damage:

I choose not to put my soul in jeopardy. There is more than what is on this earth, and I have the right to be protected from an act that may harm or damage the wellbeing of my spirit.

For others, religious beliefs complicated their stance on both MAiD and their faith. For instance, one nurse chose to participate in MAiD but not talk about it in church settings:

My priest told me [MAiD] was murder and if I was present, I was part of a murder. I’m on the fence because I’ve seen great deal of suffering. I don’t see it as somebody choosing to do harm, but as being merciful. How can God say that’s wrong?

### Living Well and Dying Well

Often interwoven with palliative care principles of neither hastening death nor prolonging life were beliefs that spoke to something much broader than a professional stance: reflections on what constitutes a good life, the sacredness of time, and insights on how to die well. One participant felt that death was not something that should be controlled:

We live in an impatient society. I see the value of having that time, however long it takes, to die.

Some participants worried that MAiD robbed people of the opportunity to experience the positive aspects of the dying process for themselves, and for their families:

The crux of palliative care is realised at the end of life. Did we navigate this journey well? I see MAiD as interrupting the opportunity to experience that, to have family be with you and enrich your life. To learn from that journey.

For some participants, it was clear that insights from their palliative care practice had a direct impact on their personal beliefs, priorities, and perspectives:

People say to me, ‘how can you do this kind of work [in palliative care]? It must be so depressing!’ And I tell them that it’s one of most rewarding and uplifting career changes I’ve ever made. It’s about family and living and celebrating life bravely.

### B) Relational Considerations

We reviewed how the philosophy of neither hastening death nor prolonging life was present in participants’ personal beliefs, impacting their professional approaches to care. Here we explore relational considerations: the impact on nurses’ therapeutic relationships, implications for group identity as palliative care specialists, and societal perceptions of palliative care.

### Walking the Journey

Palliative care nurses felt they had expertise to normalize dying and help patients achieve a good death. It is not surprising that therapeutic relationships were the bedrock on which their work with patients and families rested. Participants described their approach to as accompaniment during a most critical moment:

We aren’t hastening or prolonging; we are walking the journey alongside the patient.

This is their journey, their choice. I’m not choosing, I’m not giving the medications, I’m not making it happen, I’m basically along for the ride.

Describing their role as that of a guest in the patient’s journey did not imply passivity; rather, it reflected restraint from directing decisions regarding MAiD, while honouring patient experiences and decisions. Nurses remained actively engaged through presence, support, and counsel while exploring patient wishes:

We’re not encouraging people to go towards MAiD. It’s about quality, not quantity. Our role in MAiD is counselling people: what is your plan, what do you want, what is available? I don’t see it as hastening death because we are not involved in MAiD. I see it as exploring options. MAiD is one option out there, not necessarily what we do.

The above narrative combined restraint from directing care decisions with an active engagement in exploring MAiD as an option alongside patients. In doing so, it softened the perception of MAiD as incompatible with palliative care values. A focus on relationships, attunement, and accompaniment shifted the focus away from MAiD, emphasizing the breadth of palliative care practice:

People stop eating, they take off their oxygen, they choose to do that. Nobody looks at that as something we did. Because that’s a choice they make to hasten their death. These things happen all the time and people should have the choice to determine what treatments they get. Same as with chemotherapy or radiation, we’re not the people actively giving that. It’s an option for our clients. Our mandate is symptom management and comfort care and quality of life. Not about the actual MAiD procedure. It’s much broader than that.

### Group Identity

Not hastening death was more than an individual value; it was also central to the group identity of palliative care professionals:

I don’t feel palliative care should be involved with [MAiD] because that’s a hastened death. It goes against the definition of what we do [in palliative care], the core principle that I’ve hung my hat on.

The principles of neither hastening death nor prolonging life solidified group identity for palliative care specialists, whose values originated in the modern hospice movement. Adhering to those principles by taking a stand against MAiD was a move towards protecting group identity. Some participants felt that boundaries should exist between palliative care and MAiD not only because they felt the two were fundamentally different, but also to maintain the integrity of the speciality:

Palliative care and MAiD are two different and separate things. I value the benefit of palliative care and advocate everyday with every patient. That’s where I struggle with MAiD. It is not something that shares space with palliative care.

With group identity threatened, some members responded by tightening control over the group’s boundaries. When faced with the perception that professional identity was in jeopardy, criteria for group belonging were reinforced. One nurse who supported MAiD as an option in palliative care recounted an instance of policing boundaries that caused her to question her belonging within her team and speciality:

I was at a conference where [MAiD] came up, and somebody made a comment about ‘the importance of treating symptoms, giving better symptom control and not murdering people’. When people say it in [a public forum] it impacts our nursing group, our palliative care team.

### Unearthing Damaging Stereotypes of Palliative Care

Some participants argued that MAiD did not belong in palliative care because it unearthed stereotypes the speciality had gone to great lengths to dispel. Specifically, participants did not want to be perceived as a speciality that euthanizes patients:

We’re still having to explain to people what palliative care is and how we’re not responsible for hastening death and that we don’t kill them by giving them morphine. To bring MAiD alongside would be undoing all the work we’ve done.

This stereotype was more than just an image problem; participants worried that it diminished peoples’ confidence in palliative care. Separating MAiD from palliative care was an important distinction for palliative care nurses to maintain trust with patients and families, critical to trusting therapeutic relationships and ensuring palliative care settings remained a safe place for the dying:

People [need to] know there is a safe place for them. They don’t want euthanasia, they don’t have to be afraid of that, they can go to hospice because it’s separate.

One nurse who was in favour of MAiD but did not feel it belonged in palliative care, argued that MAiD need not be offered in the hospice context, as other procedures were not offered:

Since the beginning of the modern hospice movement, we do not prolong life nor hasten death. Palliative care can rest on that foundation if they choose not to perform [MAiD] and say, ‘we are a hospice palliative care unit and that is not what we do’. There are a lot of procedures not done in hospice, this is another one.

### C) Moral Coherence

When confronted with a new moral angle, the process of moral coherence helps make sense of this viewpoint in a way that is coherent with other values.^11^ This section showcases how nurses evaluated clinical situations that bear similarities to MAiD and considered the impact of different options on patients and families.

### Considering Similar Cases

When asked about the principle of not hastening death, many nurses reflected through clinical situations that bore similarities to MAiD. Here we report how nurses reflected through instances when palliative care prolonged life, when it could not adequately relieve suffering, and finally, whether MAiD was similar or different from palliative sedation.

### When Palliative Care Prolongs Life

Participants considered whether and how their stances towards ‘not hastening death’ and ‘not prolonging life’ differed. Their deliberations considered intended and unintended consequences, as well as goals of care:

We do not hasten death. But the truth is our interventions may prolong life. It’s not the prolonging that we’re uncomfortable with, it’s the hastening.

Neither hastens death nor prolongs life sounds like a contradiction, doesn’t it? We do all kinds of things to prolong life, but it goes back to the goals of care. What does a patient want? Do they want their life prolonged? A lot of people ask for things that could be harmful, like chemo or radiation, when they can no longer handle it physically.

Others reflected on how palliative approaches changed over time, with interventions from curative medicine stretching definitions of symptom management and supportive care:

When I started in palliative care, we provided symptom management, lots of opioids, and allowed for a natural death. Now I’m seeing more blood transfusions, where the intention is to promote quality of life, but it may artificially extend somebody’s life. So yes, it’s part of palliative care but it’s a grey area. If we take these words literally, we’re in fact hastening or prolonging at different times with different interventions.

Nurses’ deliberations demonstrated that hastening death and prolonging life were not morally equivalent, nor were they opposing principles. Reducing them to a simple binary, or relying on a literal interpretation missed critical context, nuance, and complexity in nurses’ ethical reflections.

### Palliative Sedation

As palliative sedation is a common intervention in palliative care, participants considered how it compared to MAiD with regards to the principle of not hastening death:

The part that gets foggy is if we run through what we can offer people and they are in the last days or weeks of life, then palliative sedation is an option. But if death is not imminent, maybe that’s why some people are choosing MAID. Maybe they feel their quality of life is compromised even though they still have time.

By describing palliative sedation as an alternative for patients who were not actively dying but not living well, this participant drew parallels to MAiD as an option for people who were not living well but did not have a reasonably foreseeable death. Although palliative sedation does not cause immediate death, another nurse argued that it did hasten death, but only in specific circumstances when patients are still able to eat and drink:

The palliative sedations I’ve been involved in have been close to the end of life. There are times where it becomes controversial because people are considering palliative sedation when they can eat and drink. You are sedating somebody who potentially has a prognosis of months, and they are going to die in a few weeks because they are not going to eat or drink. They are hastening death to an extent.

### When Symptom Relief is Not the Primary Driver

Some participants worried that patients may opt for MAiD before seeing what palliative care could do for symptom management and quality of life:

Anybody who is asking for [MAiD] is experiencing very high suffering. And if they had a consultation with palliative care, we would have things to offer, and they would not need to resort to MAiD.

This worry was compounded by the knowledge that palliative care is under-resourced in most jurisdictions. However, some participants also acknowledged cases where their most valiant efforts could not alter patients’ suffering; this was a challenging contradiction for participants:

For years we preached that if you’re giving good palliative care then people won’t want MAiD […] We are delivering stellar care in a beautiful setting. And yet there are still requests for MAiD. It’s not rolling out the way I expected. It’s almost like good care doesn’t necessarily impact their decision. They appreciate it. They want it. But that’s not the deciding factor.

### Considering Outcomes: Do the Means Justify the Ends?

Some considered the implications of palliative care facilities taking a stand against MAiD, and how that would impact patients:

It gets a little muddy. Would it make sense that an outside team could come and provide [MAiD] within the [hospice] without having to put a family through the distress of a transfer? I’m not sure. I have a lot of mixed feelings about it.

It can be difficult to have to transfer people out for MAiD. Do they feel abandoned by us? Should we do that? The delivery of medically assisted death can be complicated [for the facility]. And having to transfer patients out, that could be difficult on them.

When considering the impact on patients, nurses were at a crossroads, with professional allegiance and commitment to patients reflecting different moral positions. Those who felt MAiD aligned with their scope of practice argued that integrating it in palliative care prioritized patient autonomy. Those who felt that a line should be drawn between palliative care and MAiD tended to prioritize professional alignment that emphasized their expertise on dying well without resorting to MAiD.

## Discussion

Our data presented a range of perspectives on the role of MAiD in palliative care nursing, specifically regarding the principle of not hastening death. Participants showcased the ethical complexity of deciding whether and how to incorporate MAiD in their professional worldview and practice. Importantly, these findings do not suggest that palliative care nurses inherently object to MAiD, nor do they remain in perpetual uncertainty and moral tension. While some participants did express opposition and invoke conscientious objection, others were supportive of integrating MAiD into palliative care practice, and others still took position across this spectrum. The focus of our analysis is on how nurses navigated ethical complexity, rather than emphasizing the result of this process of reflection. To understand what the Canadian nursing profession is grappling with today regarding expanded MAiD eligibility, it is useful to first examine reflections on the principles of neither hastening death nor prolonging life shortly following MAiD legalization. We wish to remind the reader that this process of moral reflection has been ongoing since these interviews were conducted, as the Canadian nursing community has continued to grapple with the integration of MAiD as an end-of-life option. The palliative care nursing community, including these participants, may hold different ideas than they did at the time of the interviews. In the discussion below, we integrate our findings in the process of moral reflection: moral intuition, relational ethics, and moral coherence.^11^

### Moral Intuition

According to Pesut and colleagues,^11^ moral intuition, or a perception of moral rightness, is constructed by sociocultural factors and is rarely challenged, allowing people to remain coherent in their individual moral positions and those of their communities. In our data, sociocultural influences were reflected in participants for whom the doctrine of neither hastening death nor prolonging life aligned with religious values: MAiD was incompatible with beliefs in God’s authority on a person’s moment of death and participating in assisted death could inflict spiritual damage. Dismantling a socioculturally constructed moral position can be tricky; Pesut et al.^11^ suggest this can be accomplished by breaking moral rules carefully and strategically, to determine impact. In our data, we saw this strategy reflected in the nurse whose priest likened MAiD to murder. The nurse tried to reconcile their faith with the suffering they had witnessed in their clinical practice: they navigated this liminal place (‘on the fence’) by separating personal and professional realms. By continuing to participate in MAiD while not speaking of this in their faith community, this nurse carefully broke the rules, preserving faith-based relationships, while exercising professional moral agency.

In morally conflicting situations, another option is to only use arguments that justify one’s original position.^25,26^ With the outcome already pre-determined, argumentation reinforces allegiance to one’s intuition, allowing individuals to remain harmonious with those around them.^11^ This was exemplified in narratives of society as impatient and afraid of death; the principle of not hastening death positioned palliative care as the antidote. While this offers an appealing narrative, it excludes patients whose suffering is not alleviated by palliative care. This could stem from refractory symptoms, or a more fundamental values difference between what palliative care is designed to provide and deeply held personal beliefs about what it means to die well. We are reminded of the words of Gloria Taylor, one of the original plaintiffs in the court case that eventually resulted in Canada’s legalization of MAiD. She had carefully considered, and rejected, the scenario where she might die while receiving palliative sedation: ‘While I appreciate that others may feel differently about it, personally, I find the idea of [palliative] sedation repugnant.^30,31^

### Relational Ethics

For Pesut and colleagues,^11^ relational ethics considers the consequence on one’s relationships of taking one moral position over another. This aligns with Ferrell and Coyle’s^32^ work on the role of nurses in suffering: witnessing suffering and distress, the importance of presence and attunement to understand and respond to suffering. This ethic was apparent in our data, particularly in the theme of ‘walking the journey.’ Nurses described their role as supporting patients as they moved towards death, emphasizing accompaniment (being present) that put patient goals at the forefront. In some narratives, a focus on patient wishes seemingly avoided the discourse of hastened death altogether.

As Pesut et al.^11^ remind us, when clinical choices reflecting antithetical beliefs arise, nurses must decide whose values prevail in the therapeutic relationship. Whereas some participants focused on patient values, others reflected on how participating in MAiD would impact palliative care group identity. Pesut and Thorne^21^ noted that differing perspectives on MAiD are polarizing, significantly impacting personal and work relationships. Participants exemplified a policing of palliative care’s boundaries in response to the legalization of MAiD, with assertions that the two should remain separate. This prompted nurses to question their belonging in a speciality that did not represent their positions, and whether differing moral stances would impact professional relationships.

Beyond group identity were concerns about how palliative care was viewed by the broader community, a concern echoed in other Canadian studies.^10,17^ Participants worried that participating in MAiD would undo decades-long efforts to dispel damaging stereotypes that brought distrust and an avoidance of palliative care. Before MAiD was legalized, participants felt they could unequivocally dispel the myth that palliative care did not hasten death; after legalization, making this argument and earning patients’ trust had become more complicated.

### Moral Coherence

Moral coherence is the process one engages in, once confronted with a new moral angle, to make sense of it in a way consistent with other moral values.^11,25,26^ Participants considered palliative care’s commitment to neither hastening death nor prolonging life through comparisons with other stances they held: were instances when palliative care prolonged life similarly problematic? Was palliative sedation fundamentally different from MAiD? What about when palliative care did not alleviate suffering in ways patients found acceptable?

Despite the principles of intending to neither hasten death nor prolong life occupying an important place in palliative care’s mission and philosophy, a literal interpretation of either one missed context and nuance. Participants noted inconsistencies in how both principles were articulated and applied and reasoned by integrating other elements: the doctrine of double effect, the importance of intent, and returning to patient goals and wishes.

Palliative sedation was where things got ‘foggy:’ as a treatment for intractable suffering, it could hasten death. Participants emphasized that palliative sedation was used when other symptom management strategies failed to relieve suffering. It was perceived as an exceptional measure considered under specific circumstances. One participant questioned the ethical permissibility of palliative sedation when a patient could still eat and drink, suggesting they did not meet criteria for intractable suffering. These reflections also hinted at the challenges reported by Downie & Chandler^33^ on what constituted a reasonably foreseeable death: the ability to eat and drink suggested that death was not foreseeable neither in temporal proximity nor by indicating a predictable cause.

The subjective nature of suffering, the distress of iatrogenic suffering, the philosophical impossibility of knowing the suffering of another, and the distinction of what is tolerable and what is not, made striving for moral coherence thorny for nurses with diverse viewpoints regarding MAiD.^33,34^ Knowing that some patients requested MAiD despite receiving excellent palliative care undermined the assumption that poor access to palliative care explained interest in assisted death.

Participants stood at a crossroads between allegiance to a longstanding palliative care ideology central to their personal, professional, and moral identity,^22,23,35^ and cognizance that MAiD reflected some patients’ end-of-life preferences, overlapping with palliative care principles.^10^ In considering similarities with palliative sedation and cases where patients requested MAiD despite receiving palliative care, participants confronted new moral angles and considered whether these constituted a reasonable exception to the norm. Participants engaged their moral imagination across different scenarios to arrive at moral coherence.^11^ Finally, participants considered the implications for patients requesting MAiD in settings where it was not offered. These procedural reflections tested new moral dilemmas, such as the ethical and relational consequences of declining to provide an intervention that patients had a legal right to request. They also exposed challenges in nurses’ moral obligations, in a context where legislation seemed to run countercurrent to their professional identity and ideology.

## Limitations and Future Directions

While this study provides valuable insights, it must be situated within a specific context. Our interviews captured perspectives from early years following MAiD legalization in Canada. While these are invaluable for capturing the historical context of our current landscape, palliative care nurses currently face many new considerations. Since these interviews, MAiD eligibility has expanded to include patients whose death is not reasonably foreseeable, those who wish to make advance requests, and people for whom mental health disorders are the sole underlying medical condition. The moral reflections that our participants shared regarding intractable suffering, the limits of symptom management, and palliative sedation provide context and foreshadowing for these debates. What we do not yet know is the extent of how these legislative changes, current and future, will further stretch the moral coherence of the palliative care nursing community. Recent literature highlights how practice has evolved after a decade of MAiD integration, with clinicians engaging in complex deliberations on new issues. These include integrating MAiD in advance care planning, challenges with waivers of final consent, and patients for whom death is not reasonably foreseeable.^36^ MAiD discussions and decision-making occurring in clinician-patient-family triads also bring new questions and considerations.^37^ While our study deepened understandings of individual nurses’ moral reflection, others have examined the impact on professional practice and team-level developments such as enhanced interprofessional support.^38^ Examining the intersection of individual reflections and team-based ethical deliberations would add rich detail to this literature. As MAiD eligibility continues to expand in Canada and internationally, the nursing community will need to revisit and continue supporting ethical reflections for nurses navigating these evolving relational and moral landscapes.

## Concluding Remarks

Nurses are experts in having difficult conversations with patients and families, and discussions about MAiD are increasingly recognized as part of nurses’ scope of practice and skillset.^36^ These difficult conversations must also occur amongst clinicians within and across disciplines. When encountering dilemmas that require consideration of new moral angles, Pesut and colleagues^11^ emphasize that nurses need to engage in deliberation to ensure they are satisfied with their clinical and moral judgements. This may include moral intuition, relational ethics, and moral coherence. A good place to start is reflecting on one’s principles and dialoguing with others with similar and diverging viewpoints. Professional values add to the conversation, but do not supersede legal frameworks on patient autonomy and rights to care. Conscientious objection remains a recourse for nurses whose moral beliefs conflict with assisted death; the same can be said for the opposing moral stance of conscientious participation.^36,39^ Exploring moral coherence across similar cases can expose inconsistencies in moral reasoning or tensions requiring additional reflection.

It is also possible that deliberations will show that the philosophy of neither hastening death nor prolonging life does not hold the same moral weight in 21^st^ century palliative care; that it is a partial, but incomplete characterization of what matters most. This narrative, and the rhetoric surrounding opposition to MAiD has been loud, making it nearly impossible to consider other perspectives. Other principles, such as a holistic view of the person, living fully until death, supporting patients in defining and achieving their goals, may appear more pertinent. It is not a one-sized solution: even within clinical specialities, there is rarely consensus. Remaining attentive to a plurality of viewpoints allows nurses to engage with complex issues and ensure that all perspectives are considered. Recognizing the historical baseline of these moral reflections is an important first step to understand our current climate and support nursing communities internationally who are beginning to navigate new options for end-of-life care. Moral coherence is not an end goal, but a continuous process that requires support and engagement for clinicians and their professional communities.

## Data Availability

Due to the sensitive nature of the topic, the authors elect to not share the data.*
